# Seroprevalences and factors associated with HBV, HCV and HIV infections in haemodialysis patients in rural areas of Gabon

**DOI:** 10.4314/gmj.v59i4.11

**Published:** 2025-12

**Authors:** Martial H Ekomy, Alda M Ngoubadjambo, Edmery M Ntsounga, Herman Begouabe, Laetitia P A Ekouaghe, Moussa Togola, Léina O M Moubeyi, Cédric S Obiang

**Affiliations:** 1 Occupational Health Department, Polyclinic of Haut-Ogooue, B.P.20033, Evêché, Franceville, Gabon; 2 Franceville Haemodialysis Centre, Amissa Bongo University Hospital, B.P.150, Franceville, Gabon; 3 Public Health Department, Amissa Bongo University Hospital, B.P.150, Franceville, Gabon; 4 Biology Department, Polyclinic of Haut-Ogooue, B.P.20033, Evêché, Franceville, Gabon; 5 Department of Paediatrics, University of Health Sciences, B.P.4009 Libreville, Gabon; 6 Department of Gynaecology, Amissa Bongo University Hospital, B.P.150, Franceville, Gabon; 7 Biochemistry Research Laboratory, Masuku University of Science and Technology, B.P. 943, Franceville, Gabon

**Keywords:** Hepatitis, Seroprevalence, Haemodialysis patients, Franceville

## Abstract

**Objective:**

To determine the seroprevalence and risk factors associated with hepatitis B (HBV), hepatitis C (HCV) and human immunodeficiency virus (HIV) infection in rural haemodialysis patients at the Franceville haemodialysis centre in Gabon.

**Design:**

Retrospective, descriptive and analytical study using exhaustive sampling.

**Setting:**

Study conducted in a rural setting at the Franceville haemodialysis centre.

**Participants:**

One hundred nineteen records of haemodialysis patients in whom HBV, HCV and HIV serologies were performed. Four incomplete files were excluded.

**Main Outcome Measures:**

Viral infection, frequency of socioclinical characteristics, and univariate analysis.

**Results:**

One hundred and fifteen haemodialysis patients were included, 70 men and 45 women. The mean age was 46.64±18.74 years. The seroprevalence of infection was 1.74%, 4.35% and 12.17% for HBV, HCV and HIV, respectively. HBV infection was associated with previous dialysis (p=0.000; OR: 36.667; 95% CI [1.825-736.304]), duration of dialysis (p=0.019; OR: 0.055; 95% CI [0.003-11.324]), elevated transaminases (p=0.000; OR:112; 95% CI [3.738-3355.535]), and initial nephropathy (p=0.0157; OR:15.149; 95% CI [0.853-268.535]). HCV infection was associated with elevated transaminases (p=0.001; OR: 27.25; 95% CI [1.432-518.553]) and incident kidney disease (p=0.039; OR: 8.916; 95% CI [0.751-105.791]). HIV infection was associated with incident kidney disease (p=0.000; OR:6.852; 95% CI [1.982-23.689]),

**Conclusion:**

A low seroprevalence of HBV, HCV and a moderate seroprevalence of HIV infection has been reported in haemodialysis patients. Certain factors are associated with these infections. Systematic vaccination against HBV, responsible sexual behaviour and current and future strict hygiene measures will help to reduce viral infections in dialysis.

**Funding:**

None declared

## Introduction

Severe infections account for 50% of mortality in haemodialysis patients.^1^ The rapid immunosenescence of these patients is due to chronic kidney disease, which leads to immunodeficiency in these patients.^2^,^3^ This contributes to their high susceptibility to infection. The implementation of preventive measures against viral infections, in particular hepatitis B (HBV), hepatitis C (HCV), and human immunodeficiency virus (HIV), in haemodialysis centres remains a major challenge, especially in developing countries. These viral infections increase morbidity and mortality among in haemodialysis patients.^4^,^5^ Preventive measures include systematic vaccination against HBV, rigorous hygiene measures for HCV, responsible sexual behaviour and the use of condom for HIV.^6^,^7^

Systematic screening for these viral infections before transfusion, in blood donors and in patients with renal failure before dialysis, has resulted not only in a reduction in contamination, but also in a reduction in the seroconversion of these viral infections in haemodialysis patients.^8^ However, studies of haemodialysis patients have highlighted nosocomial infections as a factor that may predispose these patients to viral infections. Prevention of infectious diseases in haemodialysis patients also includes strict monitoring of compliance with hygiene measures and rigorous asepsis by all haemodialysis unit staff.^8^,^9^

In Gabon, National policies for hepatitis and HIV/AIDS prevention and care are accessible to both at-risk populations and the general population. Several actors are involved in the fight against HIV/AIDS and hepatitis: the public sector, civil society organisations and development partners. Awareness-raising and screening campaigns are organised, and outpatient treatment centres are set up to care for infected patients throughout the country. However, in our rural context, diagnosis and treatment of hepatitis remain prohibitively expensive, both for the general population and for haemodialysis patients. There are eleven haemodialysis centres, four of which are located in the interior of the country, two of which are public: Port-Gentil and Franceville. The latter serves the entire population of the south-eastern region of the country. Diagnosis and treatment of patients infected with hepatitis B and C viruses and HIV remain difficult in rural areas, and can contribute to morbidity and mortality in haemodialysis patients. Problems with the supply of consumables, medicines and medical equipment, leading to rapid stock-outs in both hospitals and pharmacies. The regular use of traditional medicine (which is poorly supervised by the authorities) and the low socio-economic status of the population mean that haemodialysis patients in rural areas are at very high risk of viral infectious morbidity and mortality. Studies carried out in and around Franceville have shown that hepatitis B and hepatitis C virus infections and Human Immunodeficiency Virus circulate at relatively low to moderate levels.^10^,^11^ Rural areas, particularly Haut Ogooué and Ogooué Lolo (in the south-east of the country) remain areas of high HCV prevalence.^10^ In Koulamoutou, in the province of Ogooué Lolo, between 2012 and 2017, the seroprevalence of HCV antibodies was 6.2%, the seroprevalence of HBsAg was 5.9%, and the seroprevalence of HIV was 3.1%. 12 In addition, a study conducted in Franceville, in the province of Haut Ogooué (study site), found a seroprevalence of HBsAg of 9.3% and a seroprevalence of hepatitis C infection of 8.8% among patients living with HIV.^13^ These studies are leading to research being carried out in rural areas, among haemodialysis patients who are a vulnerable population and whose care requires all these epidemiological data to be taken into account. Studies have been carried out in urban areas, in Libreville (the country's capital), among haemodialysis patients. These studies confirmed the transmission of the hepatitis C virus during chronic haemodialysis, with an incidence rate of 15.1 cases per 1,000 person-years. They also confirmed the risk factors of long-term haemodialysis and the use of transfusions for infection with the hepatitis C virus.^14^ They highlighted the high risk of HCV/HBV seroconversion in medical facilities outside the university hospital centre (CHU).^15^ However, no study has been conducted among haemodialysis patients in rural areas.

We therefore set out to determine the seroprevalence and factors associated with hepatitis C, hepatitis B, and human immunodeficiency virus infections among rural haemodialysis patients at the Franceville Haemodialysis Centre in Gabon.

## Methods

### Description of the study site

Haut Ogooué is the second largest province in the country. Franceville, the province's capital, has a population of 110,568 (2013 census) and covers an area of 40,000 km^2^, or 1/6 of the country's territory. The Franceville haemodialysis centre has been operational since October 2021. It is an autonomous unit serving the entire population of the south-east of the country. It has a capacity of 10 beds, 10 operational dialysis machines (Fresenius 4008S), two water treatment rooms, one generator and one inverter. The medical staff comprises 16 permanent employees, including two nephrologists, nine nurses, one manager, one secretary, one biomedical technician, one hygienist, one surface technician, and three trainees.

### Type and duration of study

We conducted a retrospective descriptive and analytical study over a two-year period, from October 20 2021, to December 31 2023. All medical records of patients hospitalised at the CHUAB haemodislysis centre in Gabon during the study period were included.

### Study population and sample

The study population consisted of all haemodialysis patients treated for renal failure at the Franceville Haemodialysis Centre during the study period. This is a population of 202 renal failure patients.

### Inclusion and non-inclusion criteria

All records of patients with chronic renal failure on haemodialysis who had viral serology for hepatitis C, B, and HIV, and viral RNA testing for HCV by PCR, performed at the CHUAB Haemodialysis Centre during the study period, were included in the study. Thus, 119 files were eligible for the study (the total population of haemodialysis patients at the centre at the time of the study). Our sample consisted of 115 medical records, as four records were excluded from the study due to insufficient information.

### Determining the sample size

The minimum sample size for the study was calculated using the Cochran formula.^16^ A confidence level of 1.90, a standard deviation of p of 0.5 and a margin of error of 10% were assumed. This gave a minimum sample size of 96. Assuming a non-response rate of 10% a final sample size of 107 was determined for the study.

### Methods and techniques for collecting data

Data were collected using a questionnaire that included socio-professional data (age, sex, nationality, place of residence), clinical data (medical history, HBV vaccination, transfusions received), and biological data (anti-HCV antibodies, anti-HIV antibodies, Hbs antigen, transaminases), obtained from patients' medical records. All viral serologies were performed using rapid diagnostic tests: Hbs antigen was detected by a third-generation Murex HBsAg enzyme immunoassay (EIA, Mediff, Aubagne, France) (sensitivity/specificity: 98.94% (95% CI)/99.0%(95% CI)), detection limit (<10IU/ml). Anti-HCV antibodies were detected using the ImmunocombII HCV kit (Alere S.A.S. Jouy En Josas, France) (sensitivity/specificity: 67.5%(95% CI)/100%(95% CI)), detection limit (10- 15IU/ml). Anti-HIV-1 & 2 antibodies were detected by a highly sensitive enzyme immunoassay (EIA) test kit using Murex HIV Ag/Ab Combination (EIA 1, Mediff, Aubagne, France) (sensitivity/specificity:100%(95% CI)/99.0%(95% CI)), detection limit (2.5-6.5pg/ml). Confirmatory testing was performed using the ImmunocombII HIV-1 & 2 Bispot Test (Orgenics, Yavne, Israel) (sensitivity/specificity:100%(95% CI)/98.9%(95% CI)), detection limit (6- 32pg/ml).

Due to high cost and our rural setting, only one of our patients was tested for HCV viral RNA by PCR (polymerase chain reaction). Plasma RNA was extracted using the QIAamp viral RNA mini kit (Qiagen, Courtabœuf, France). RT-PCR followed by nested PCR was performed using primers targeting the 5′UTR region, with a detection threshold of 1000 IU/mL, to amplify a 262 bp.^17^,^18^ All of these tests were performed according to the manufacturer's recommendations.

Biochemistry (transaminases) was performed on a HUMASTAR100 instrument (reagent: GPT(ALAT) IFCC mod. LiquiUV, GOT(ASAT) IFCC mod. LiquiUV). Transaminase levels were considered normal for alanine aminotransferase (ALAT) levels between 10IU/L - 35IU/L and aspartate aminotransferase (ASAT) levels between 10IU/L - 40IU/L. Liver biopsy is not a common practice in our rural health facilities. Patients are systematically referred to the capital city, but due to limited financial resources, they do not attend.

### Data entry and analysis

Data were analysed using Epi Info 7.2.6 software. Descriptive results were expressed as numbers and percentages for qualitative variables. Quantitative variables were described by calculating the mean, standard deviation and extreme values (minimum and maximum). Tables and graphs were generated using Microsoft Office Excel 2016 (Microsoft Corporation, Redmond, WA, USA). Bivariate analysis was used to highlight the relationships between the variables. Given the small sample sizes, Fisher's exact test was used for the qualitative variables. For quantitative variables, the non-parametric Kruskal-Wallis test was used to compare distributions between groups, with a significance threshold of 5% (p=0.05). Logistic regression analysis was performed to search for associations between the dependent variable and a putative predictor variable, and the \odds ratio (OR) and the 95% confidence interval (95% CI) were calculated.

### Ethical considerations

The respect and dignity of all study participants were maintained. An anonymous questionnaire was used. The work described did not involve experiments on patients, human subjects or animals. The study was approved by the Medical Committee and the General Management of CHUAB. All the tenets of the Declaration of Helsinki regarding human subjects in research were followed during the data collection process. Ethical approval for the study was granted by the Regional Ethics Committee of the province of Haut-Ogooué under the reference number PROT N°02/2021/MSAS/DRSSEF.

## Results

### Descriptive results

Of the 202 patients with renal failure hospitalised and monitored in the haemodialysis unit during the study period, 119 received haemodialysis, representing a prevalence of 58.91%. We collected records for 115 of the 119 haemodislysis patients, yielding a participation rate of 96.64%. Men represented 60.87% (n=70), and women represented 39.13% (n=45). The male/female sex ratio was 1.56. The mean age was 46.64±18.74 years, with extremes of 5 and 79 years. The mean duration of dialysis was 7.86±18.08 months, ranging from 0.5 months to 96 months. Among the 115 chronic haemodislysis patients, the seroprevalence of viral infections was 18.26% (n=21). Five patients tested positive for anti-HCV antibodies, including one by PCR, resulting in a seroprevalence of 4.35%. Only 1 in 7 patients (14.29%) with viral hepatitis were receiving treatment. No patient in the sample had a viral co-infection. Of the two patients with hepatitis B infection, neither had been vaccinated against HBV. Only one haemodislysis patient in our study had been tested for HCV RNA by PCR.

This was a case of seroconversion of HCV infection. The mortality rate in our study was 40.87%, of which 8.51% were haemodialysis patients with viral infection. The socio-professional and clinical characteristics of the sample are summarised in Table 1.

**Table 1:** Socio-professional and clinical characteristics of the sample

		Frequency
		n (%)
**Items**	<=20	14 (12.17)
	>20 - 35	18 (15.65)
**Age**	>35 - 50	26 (22.61)
	>50	57 (49.57)
**Sex**	Female	45 (39.13)
	Male	70 (60.87)
**Nationality**	Gabonese	108 (93.91)
	Other	7 (6.09)
**Occupation**	Worker	40 (34.78)
	Unemployed	75 (65.22)
**City**	Franceville	30 (26.09)
	Mouanda	36 (31.3)
	Koulamoutou	6 (5.22)
	Boumango	1 (0.87)
	Other	42 (36.52)
**Alcohol**	Yes	9 (7.83)
	No	106 (92.17)
**Viral infection status**	Hepatitis B	2 (1.74)
	Hepatitis C	5 (4.35)
	HIV	14 (12.17)
	Negative	94 (81.74)
**Hepatitis B vaccination**	Yes	11 (9.57)
	No	104 (90.43)
		
**Haemodislysis duration per month**	<=1	56 (48.69)
	>1 - 12	41 (35.65)
	>12 - 36	13 (11.30)
	>36	5 (4.34)
		
**Initial nephropathy**	Toxic	8 (6.96)
	Diabetic	13 (11.3)
	Sickle cell	1 (0.87)
	Dysthyroidism	1 (0.87)
	Hypertensive	37 (32.17)
	Undetermined	3 (2.61)
	Infectious	38 (33.04)
	Lithiasis	1 (0.87)
	Lupic	3 (2.61)
	Myelomatosis	1 (0.87)
	Neoplastic	3 (2.61)
	Obstructive	2 (1.74)
	Kidney polycystosis	2 (1.74)
	Cardiorenal syndrome	2 (1.74)
**Transfusion**	Yes	62 (53.91)
	No	53 (46.09)
	Heart disease	5 (4.35)
**Antecedent**	Diabetes	35 (30.43)
	CH	4 (3.48)
	HBP	17 (14.78)
	NO	54 (46.96)
**Evolution**	Deceased	47 (40.87)
	Recovery	14 (12.17)
	Dialysis stopped	12 (10.43)
	CKD	17 (14.78)
	CH	25 (21.75)

CKD: chronic renal failure, CH: chronic haemodislysis, HBP: high blood pressure.

### Analytical results

Univariate and multivariate logistic regression were used to identify predictive risk factors for hepatitis B and C virus infection and human immunodeficiency virus infection. Univariate analysis showed that the predictive risk for hepatitis B virus infection was: initial kidney disease, elevated transaminases, duration of dialysis, and history of dialysis (see Table 2).

**Table 2:** Risk factors for HBV infection in chronic haemodialysis patients in univariate and multivariate analysis

	Total number	Positive	Univariate analysis		

		n (%)	p-value	OR	95% CI
**Age**					
**>20 – 35**	18	1 (5.56)	0.177	5.647	0.336-94.686
**>50**	57	1 (1.75)	0.991	1.018	0.062-16.676

**Antecedent**					
**CH**	4	1 (25)	0.000	36.667	1.825-736.304
					
**Transfusion**					
**YES**	62	2 (3.23)	0.187	ND	ND

**Vaccination**					
**NO**	104	2 (1.92)	0.689	0	ND
					
**Haemodialysis duration per month**					
**<=1**	56	1 (1.69)	0.970	1.055	0.064-17.277
**>1 - 12**	41	1 (2.43)	0.019	0.055	0.003-11.324
**Evolution**					
**Deceased**	**47**	1 (2.13)	0.0696	1.456	0.088-23.883
**Recovery**	14	1 (7.14)	0.098	7.692	0.453-130.539
**Transaminase elevation**					
**Significant cytolysis**	**4**	1 (25)	0.000	36.667	1.825-736.304
**Light cytolysis**	**2**	1 (50)	0.000	112	3.738-3355.535
**Nephropathy**					
**Toxic**	**8**	1 (12.5)	0.0157	15.149	0.853-268.535
**Hypertensive**	**37**	1 (2.7)	0.586	2.138	0.130-35.172

**Nationality**					ND
**Gabonese**	106	2 (1.85)	0.716	ND	
**Occupation**					ND
**Without**	75	2 (2.67)	0.297	0	ND

OR: Odds Ratio; CI: confidence interval; CH: chronic haemodislysis

Univariate analysis of hepatitis C viral infection revealed initial nephropathy and elevated transaminases as predictive risk factors (see Table 3). For human immunodeficiency virus (HIV) infection, univariate analysis revealed initial kidney disease as the only predictive risk factor (see Table 4). Multivariate analysis revealed no predictive risk factors for either hepatitis B, C or human immunodeficiency virus infection.

**Table 3:** Risk factors for HCV infection among chronic haemodialysis patients in univariate and multivariate analysis

Item	Total number	Positive	Univariate analysis		

		n (%)	p-value	OR	95% CI
**Age**					
**>35 - 50**	26	1 (3.85)	0.886	0.851	0.091-7.954
**>50**	57	4(7.02)	0.164	4.302	0.465-39.729
**Haemodialysis duration per month**					
**<=1**	56	3(5.36)	0.605	1.613	0.259-10.035
**>1 - 12**	41	1(2.44)	0.454	0.437	0.047-4.051
**>12 - 36**	13	1(7.69)	0.599	0.571	0.068-4.759
**Evolution**					
**Deceased**	47	3(6.38)	0.791	2.250	0.361-14.018
**Recovery**	14	1(7.14)	0.584	1.865	0.193-17.993
**Transaminase elevation**					
**Significant cytolysis**	4	3(75)	0	163.5	11.431-2338.576
**Light cytolysis**	2	1(50)	**0.001**	27.25	1.432-518.553
**Normal**	109	1(0.92)	0.903	ND	ND
**Initial nephropathy**					
**Infectious**	37	2(5.41)	0.701	1.428	0.228-8.938
**Diabetic**	13	1(7.69)	0.604	0.558	0.060-5.188
**Neoplastic**	1	1(100)	0.000	ND	ND
**Undetermined**	3	1(25)	**0.039**	8.916	0.751-105.791
**Nationality**					
**Gabonese**	108	5(4.63)	0.561	ND	ND
**Occupation**					
**Worker**	40	2(5)	0.802	1.263	0.202-7.889
**Without**	75	3(4)	0.803	1.261	0.144-8.813

OR: Odds Ratio; CI: confidence interval

**Table 4:** Risk factors for HIV infection among chronic haemodialysis patients in univariate and multivariate analysis

	Total number	Positive	Univariate analysis		

		n(%)	p-value	OR	95% CI
**Age**					
**<=20**	14	1(7.14)	0.539	0.521	0.063-4.319
**>20 – 35**	18	3(16.67)	0.525	1.563	0.389-6.274
**>35 – 50**	26	5(19.23)	0.211	2.116	0.641-6.986
**>50**	57	5(8.77)	0.268	0.524	0.164-1.671
**Antecedent**					
**Diabetes**	35	4(11.43)	0.871	0.903	0.263-3.103
**Transfusion**	62	6(9.68)	0.375	0.602	0.195-1.864
**CH**	4	1(25)	0.424	0.513	0.243-25.982

**No History**	54	9(16.67)	0.319	ND	ND
**Haemodialysis duration per month**					
**<=1**	56	7(12.5)	0.917	1.061	0.347-3.246
**>1 - 12**	41	5(12.2)	0.449	1.003	0.312-3.221
**>12 - 36**	13	1(7.69)	0.599	0.570	0.068-4.759
**>36**	5	1(20.00)	0.729	1.476	0.159-13.651
**Evolution**					
**Deceased**	47	8(17.02)	1.746	2.119	0.683-6.573
**Refusal of dialysis**	12	1(8.33)	0.667	0.629	0.075-5.286
**Recovery**	14	3(21.43)	0.258	2.231	0.538-9.249
**Initial Nephropathy**					
**Infectious**	37	10(27.03)	**0.000**	6.852	1.982-23.689
**Diabetic**	13	1(7.69)	0.599	0.570	0.068-4.759
**Hypertensive Nationality**	37	3(8.11)	0.358	0.537	0.141-2.056
**Gabonese**	108	13(12.04)	0.860	0.821	0.091-7.372
**Other**	7	1(14.29)	0.861	0.822	0.109-20.409
**Occupation**					
**Worker**	40	6(15)	0.498	1.478	0.474-4.603
**Without**	75	8(10.67)	0.500	1.473	0.444-4.691

OR: Odds Ratio; CI: confidence interval, CH: chronic haemodislysis

## Discussion

Determination of the seroprevalence and factors associated with HBV, HCV, and HIV infections at the Franceville haemodialysis centre in Gabon revealed high rates of seroprevalence and numerous associated factors, particularly duration of dialysis and pre-existing kidney disease.

### Seroprevalence of HBV/HCV infection

The seroprevalence of HBV infection (HBsAg) in this haemodialysis population was 1.74%. HCV infection was 4.35%. The seroprevalence rates observed in our study are lower than those seen in the general Gabonese population. In fact, the study by BISSEYE Cyrille et al., conducted in 2018 in the rural area of Koulamoutou, reported seroprevalences of 5.9% and 6.2% for HBV and HCV infection, respectively.^12^ Our results differ from those reported in the literature, which indicate a higher prevalence of hepatitis B virus (HBV) and hepatitis C virus (HCV) infections among haemodialysis patients than in the general population.^8^,^19^,^20^ However, these seroprevalences of HBV and HCV infection in our study may be underestimated and may in fact be higher. In fact, only one haemodialysis patient in our study was tested for HCV viral RNA by PCR. This case represented seroconversion to HCV infection that we identified. In addition, studies conducted in the Haut-Ogooué region of Gabon (our study site) and those described in the literature have shown occult HBV and HCV infections.^13^,^21^,^22^,^23^,^24^ Our results were similar to those obtained at a university hospital in Monastir, Tunisia, in 2017, where the seroprevalence of hepatitis B virus (HBV) and hepatitis C virus (HCV) infections among haemodialysis patients was 5.5% and 7.3%, respectively.^5^ However, they were much lower than the seroprevalences of HBV and HCV infections in haemodialysis patients in Egypt, which were 60.7% and 60.9%, respectively.^25^,^26^ The seroprevalences of HBV and HCV infections in haemodialysis patients vary from country to country. They are minimal in Europe and still very high in Africa, especially in sub-Saharan Africa.^27^ Poor hygiene practices, unsystematic HBV vaccination, lack of training of medical staff, frequent shortages of treatment materials, patient poverty, excessive prices of drugs used to treat viral liver infections, untimely transfusions to combat anaemia, and the rural context remain obstacles in our countries to combat HBV and HCV infections in haemodialysis patients effectively.^7^

In addition, these infections have a significant impact on the survival of haemodialysis patients.^23^,^28^ The high mortality rate of 40.87% in our study may well reflect this reality.

### Factors predictive of HBV infection

In univariate analysis, several predictive factors have been associated with HBV infection, including history of dialysis, duration of dialysis, elevated transaminases and initial kidney disease. Studies in the literature also highlight the duration of dialysis as a predictive factor for hepatotropic infections, particularly HBV and HCV.^5^,^8^,^29^ The frequent use of blood transfusions to combat anaemia may explain this association. Although in our study, blood transfusion was not associated with viral infections. In our study, 54% of haemodialysis patients were transfused. However, in several countries such as Tunisia, transfusions are being used less and less in haemodislysis patients 5 Unfortunately, in our rural Gabonese context, transfusions are still performed in haemodislysis centres because there is no alternative for the time being. Vaccination was not a predictive factor in our study.

However, we found that 90.43% of haemodialysis patients were not vaccinated against HBV, despite all the awareness campaigns carried out in the haemodialysis centre. In addition, the HBV-infected patients in our study were not vaccinated. This lack of systematic vaccination among haemodialysis patients may be partly explained by the very long stock-outs of HBV vaccine in pharmacies and vaccination centres in our rural setting.

### Factors predictive of HCV infection

HCV infection in haemodialysis patients remains a major problem in haemodialysis centres. In our study, we found a prevalence of HCV infection of 4.35%. This is higher than the figure of 2.1% found by Itoudi et al, in Libreville (the country's capital) in 2020, which was 2.1% (before dialysis). This difference can be explained, on the one hand, by a slightly lower prevalence of HCV infection in Libreville (Estuaire) than in Franceville (Haut-Ogooué).^10^ On the other hand, it can be explained by the continuous training of medical staff, more rigorous hygiene measures, the availability of treatment equipment and patients' early awareness of their disease in the capital than in rural areas. In univariate analysis, we found that elevated transaminases and initial nephropathy were predictive factors associated with HCV infection. The Libreville study found that the duration of dialysis and blood transfusion were factors associated with positive seroconversion to hepatitis C. We also found seroconversion in our sample, as in a number of studies in the literature that tested for viral RNA by PCR.^8^,^14^ This seroconversion once again highlights the possibility of the existence of occult hepatic viral infections, as described above. Therefore, the cost of PCR testing for patients with chronic diseases in our country should be significantly reduced to enable proper and comprehensive management. Only one patient was receiving treatment for viral hepatitis C, and the other four could not afford it. The care of these patients is difficult and incomplete.

### Seroprevalence and predictive factors for HIV infection

In our study, we found a seroprevalence of HIV infection of 12.17%. In the most recent Demographic and Health Survey (DHS) in Gabon, HIV prevalence was reported to be higher in rural areas (4.0%) than in urban areas (3.5%), and the overall prevalence in the province of Haut-Ogooué, in the southeast of the country, was 2.8%.^30^ Our results are therefore much higher than those found in the general population. They were also higher than those reported by Mokoli et al. (2016) in haemodialysis centres in Kinshasa (4.8%). This difference may be explained by different attitudes towards HIV prevention measures. The rural context of our study may also explain this difference. We did not find any viral co-infections in our study.

Previous studies in Gabon have reported low levels of viral co-infection between HIV, HBV and HCV.^13^,^14^,^31^ In our study, only initial kidney disease was found to be predictive of HIV infection. All our HIV-infected haemodialysis patients are regularly monitored in the outpatient treatment centre. Treatment interruptions also remain an additional problem for these patients.

Rigorous, universal and long-term hygiene measures need to be introduced or reinforced in haemodialysis centres (for all hospital staff, patients' relatives and patients). Systematic HBV vaccination of haemodialysis patients and medical staff, and responsible sexual behaviour (condom use) are necessary. Alert the relevant authorities to the need for a continuous supply of treatment equipment, consumables and drugs to haemodialysis centres in the country.

### Limitations of the study

We identified only one PCR procedure, but no liver biopsy report was available in the study files. Several patients were either deceased or lost to follow-up. It was not possible to conduct additional interviews for the four incomplete files. Our results must be interpreted with great caution because the study does not represent all haemodislysis patients in the country.

## Conclusion

The study revealed a low seroprevalence of HBV, HCV and a moderate seroprevalence of HIV infection among haemodialysis patients at the Franceville haemodialysis centre. It also identified predictive risk factors for these viral infections, particularly duration of dialysis and preexisting kidney disease.
